# Optimising data sharing whilst protecting participant privacy: a data note describing processed data from a qualitative study of healthcare professionals’ experiences of caring for women with false positive screening test results

**DOI:** 10.1080/21642850.2024.2449400

**Published:** 2025-01-12

**Authors:** Hannah A. Long, Peter Branney, David P. French, Joanna M. Brooks

**Affiliations:** a Division of Nursing, Midwifery, and Social Work, School of Health Sciences, University of Manchester, Manchester, UK; b Department of Psychology, School of Social Sciences, University of Bradford, Bradford, UK; c Manchester Centre for Health Psychology, Division of Psychology and Mental Health, School of Health Sciences, University of Manchester, Manchester, UK

**Keywords:** Qualitative data, open data, template analysis, breast screening, false-positive test results

## Abstract

**Introduction:**

The present article describes the processed data generated in a qualitative interview study and template analysis. Many women find the experience of being recalled and receiving a false-positive breast screening test result to be distressing. The interview study aimed to understand breast screening healthcare professionals’ (HCPs) experiences of providing care during the recall process and when receiving false-positive screening test results, including their communication with women around false-positive screening test results.

**Methods:**

Twelve HCPs from a single screening unit in the English National Health Service Breast Screening Programme participated in semi-structured interviews in 2020. All participants were female. A range of HCPs roles were recruited, including advanced radiographer practitioners, breast radiographers, breast radiologists, clinical nurse specialists, and radiology healthcare assistants. The data were analysed thematically using template analysis from a limited realist perspective.

**Results:**

A total of 20 data files are described, reflecting the iterative nature of template analysis. The files report various versions of codes, subthemes, themes, and every template produced during analysis. The files are publicly available on the Open Science Framework and UK Data Service (ReShare).

**Discussion:**

This data note outlines our approach to conducting a template analysis of qualitative data while protecting highly identifiable data, which is stored in a non-public archive and only available to the study team. It offers a practical, worked example of the template analysis process, thereby providing a detailed illustration beyond the concise summaries typically found in published reports, and complementing methodological papers of template analysis.

## Introduction

National breast screening programmes aim to detect and treat breast cancers earlier in order to reduce mortality and other adverse outcomes (Marmot et al., 2013). However, all screening programmes involve harms for those who are screened, such as false-positive screening test results (Marmot et al., 2013). False-positive screening test results occur when an initial breast screening test indicates an abnormality, requiring further diagnostic procedures that ultimately show cancer is not present. In the English National Health Service Breast Screening Programme (NHSBSP), approximately 3% (i.e. 54,000) of women screened annually receive false-positive screening test results (NHS Digital, 2024).

False-positive screening test results have been associated with increased breast cancer-specific anxiety and worry for women undergoing breast screening (Bond et al., 2013; Long et al., 2019b; Nelson et al., 2016), but their experiences of such test results are not well understood, particularly for women within the NHSBSP. The role of breast screening healthcare professionals (HCPs) in addressing women's negative emotions following false-positive screening results has also been unclear. Qualitative research has suggested that women appreciated clear and thorough explanations of such results, but some recalled unclear communication and experienced lingering uncertainty (Long et al., 2019). Currently, there is no specific guidance for breast screening HCPs on the communication of results beyond the requirement to deliver clear, accurate, and prompt assessments in person by a clinical team member. There has been little research with HCPs who provide care for women during this time. However, HCPs are well-positioned to provide valuable insights into women’s emotional responses to being recalled in screening and the communication of results. Therefore, the aim of the qualitative interview study, to which the present data note relates, was to understand breast screening HCPs’ experiences of providing care for women undergoing further diagnostic tests at screening assessment, including their communication with women around false-positive screening test results (Long et al., 2024b).

The present data note describes the *processed* data associated with this qualitative study, as the *raw* data is closed. Raw data refers to the unaltered, original data collected during the study (e.g. interview audio recordings and interview transcripts). In contrast, processed data is derived from raw data through analytical procedures, such as coding and theme development in a thematic analysis, and can include organised or summarised outputs (e.g. thematic frameworks) (Branney et al., 2022). Guidelines for open data sharing highlight the importance of protecting participants’ privacy (Prosser et al., 2023; National Institutes of Health, 2023; Economic and Social Research Council, 2018; NIHR Open Research, 2024; The European Parliament and of the Council of the European Union, 2019). Qualitative health psychology and behavioural science research involves a range of data and metadata that varies in the extent to which they have been processed and to which participants are potentially identifiable from their data. As researchers, we argue that our responsibilities are to protect participants’ identities and maximise their contribution to research. Challenges (and opportunities) lie in finding a way to both optimise data sharing and protect participants’ privacy.

The present data note reports the detailed procedure of conducting the (template) analysis of qualitative interview data, while protecting the highly identifiable data stored in a non-public archive and only available to the study team. We hope that the resulting data analysis files (Long et al., 2019a; Long et al., 2024a) provide a clear and richly detailed audit trail of analysis development. By clearly outlining the steps and decisions made during the template analysis process, we hope that the present data note provides a more comprehensive picture than the typically concise summaries found in published study reports (Branney et al., 2022; Norris et al., 2024).

The rationale for creating the present data note is threefold. Firstly, we aimed to increase the ‘Findability’ and ‘Accessibility’ of the processed data by producing a corresponding data note (Norris et al., 2024). This data note describes the iterative development of the analysis template, and thus supplements and supports the published study article where the findings are reported (Long et al., 2024b). Secondly, we wished to demonstrate a means of engaging with open qualitative research practices (such as open data sharing), where possible, and in circumstances where the raw dataset is closed and cannot be made publicly available. Guidelines for scientific data management recognise there are legitimate reasons for not making data open and advocate a principle of ‘as open as possible, as closed as necessary’ (NIHR Open Research, 2024; The European Parliament and of the Council of the European Union, 2019). There is a risk of participant identification in qualitative research, even when complete de-identification of transcripts is performed (Pascale et al., 2022). In our interview study, participants had particular and identifying healthcare roles. Finally, the creation of data notes to describe qualitative research datasets is still relatively uncommon in qualitative health research, with a few notable exceptions (e.g. (Willén & Granhag, 2015)). By documenting and describing our data analysis and processed data, we hope to contribute to an enabling environment for qualitative researchers applying open research practices in their work, for the wider benefit of the qualitative open science community.

## Materials and methods

The study protocol was preregistered on the OSF (Long et al., 2019a).

### Participants and setting

Twelve HCPs were recruited from a moderately sized NHSBSP screening unit in North-West England. All participants were female (Table 1). HCPs were eligible if they (a) worked at the participating NHSBSP unit, (b) provided care for women during screening assessment and/or at biopsy results appointments, (c) were sufficiently fluent in English. Participants were recruited purposively to achieve diversity in their professional roles. A range of HCPs were recruited, including advanced radiographer practitioners (n = 3), breast radiographers (n = 2), breast radiologists (n = 4), clinical nurse specialists (n = 2), and radiology healthcare assistants (n = 1).

**Table 1.** Sample demographic characteristics (n = 12).

Demographic characteristic	n (%)
Age range (years)	
25–34	3 (25)
35–44	5 (42)
45–54	2 (17)
55–64	2 (17)
Healthcare professional role	
Advanced radiographer practitioner	3 (25)
Clinical nurse specialist	2 (17)
Consultant breast radiologist	3 (25)
Breast radiographer	2 (17)
Breast radiologist	1 (8)
Radiology healthcare assistant	1 (8)
Years in current professional role	
<1	2 (17)
1–4	7 (58)
5–9	1 (8)
20–24	2 (17)

### Procedure

Two study collaborators at the unit emailed study invitations and participant information sheets to individual HCPs and participated in interviews themselves. Data were collected via face-to-face semi-structured interviews. An interview guide was developed based on prior relevant work (Long et al., 2019b) and piloted with a consultant breast radiologist in the study team. HAL conducted interviews in private meeting rooms at the unit between January and February 2020. HAL is a white British researcher of pre-screening age and undertook this study as part of her doctorate in Health Psychology at a UK university. Participants provided written informed consent and completed a short demographic questionnaire (i.e. age range, gender) and gave details of their role (i.e. job title, years in role) before their interview. Interviews were audio-recorded and transcribed verbatim by an independent party. Data were collected and stored in accordance with the Data Protection Act 2018, General Data Protection Regulation (GDPR), and the host university’s Research Data Management Policy. Participants were given pseudonyms and identifying information (e.g. personal, place, or organisation names) was removed from transcripts. This was in line with the above policies and the NHS Research Ethics Committee who gave ethical approval for the study. HAL made field notes after each interview and kept a reflexive journal throughout the research.

## Data description

The interview transcripts were analysed using template analysis (Brooks et al., 2015; King & Brooks, 2017). Template analysis is a distinctive style of thematic analysis, with key defining characteristics. While template analysis is part of a broader category of thematic qualitative data analysis approaches (which commonly involve defining and organising themes into a structure that represents their conceptual relationships), it is its own distinctive style of thematic analysis, with key defining characteristics. A novel feature of template analysis is the iterative development of a coding template. This template is often initially constructed using a subset of data and is continuously refined as it is applied to additional data. Unlike many other forms of thematic analysis, template analysis allows for the integration of inductive and deductive (‘bottom up’ and ‘top down’) coding, rather than insisting on an explicit distinction between these, and permits (but does not require) the use of a priori codes (defined in advance of coding). Additional defining features of template analysis are its flexibility and adaptability. While hierarchical coding is emphasised, template analysis does not impose a particular or fixed sequencing of coding levels and supports varied styles and formats for the coding template. Researchers using template analysis can therefore define, adjust, and reorganise themes as analysis progresses to create a nuanced and highly tailored template that captures both hierarchical and lateral relationships, reflecting the complexities of their specific research question and context (Brooks et al., 2015; King & Brooks, 2017).

A limited realist approach to inquiry was adopted as we sought to understand what is true for participants, including their personal, subjective understanding and experiences, while also producing findings of value and wider utility in applied healthcare settings (Brooks et al., 2015; King & Brooks, 2017).

**Figure 1.**
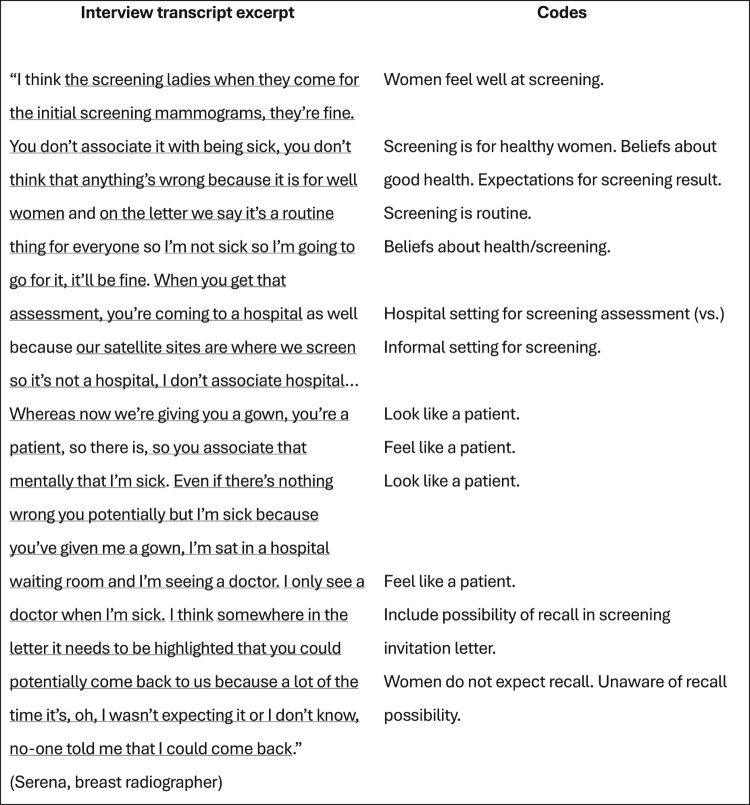
An example of preliminary coding on an extended excerpt of anonymised interview data from the first subset of interview transcripts. These codes form the basis of parts of Theme 1 within the developing template. The full list of preliminary codes can be found in archived file 1 *Preliminary coding 1* on the OSF and ReShare. This Figure has been retrospectively created for the purposes of illustrating coding within the present Data Note.

**Figure 2.**
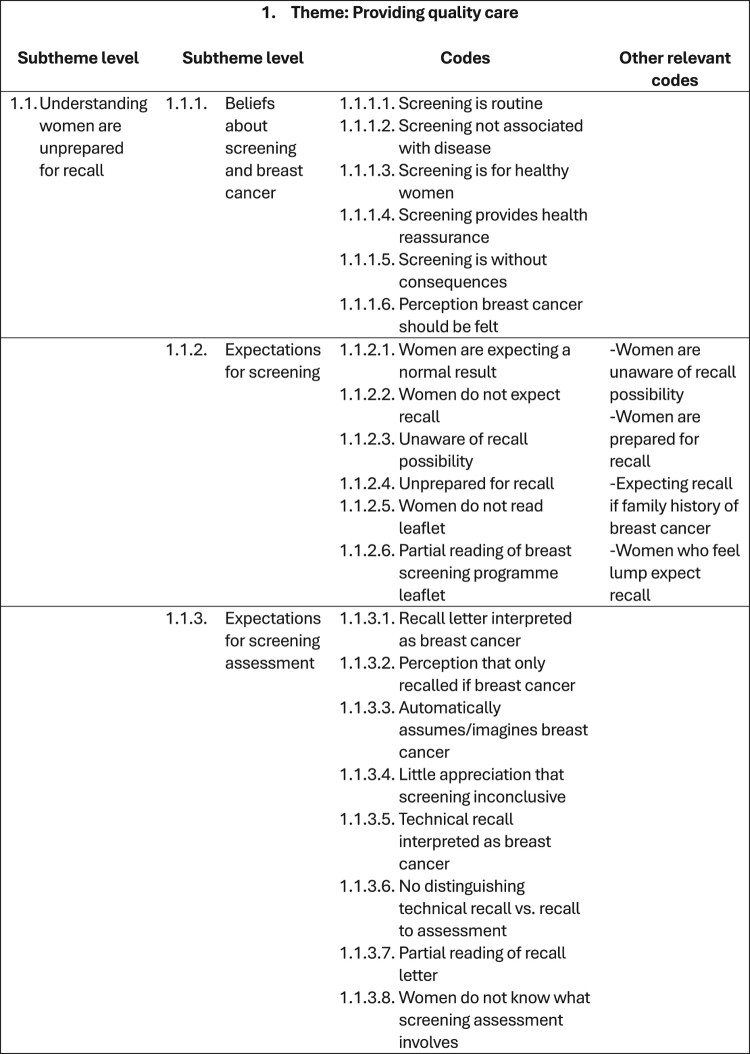
An example section of Theme 1 from the entire initial template (which includes all preliminary overarching themes and all lower-level subthemes and codes). This figure indicates initial clusters of codes and provides the names given to represent the theme and subthemes at this stage in the analysis. The full (10 page) version can be found in archived file 8 *Template 3 longer_revised* on the OSF and ReShare.

**Figure 3.**
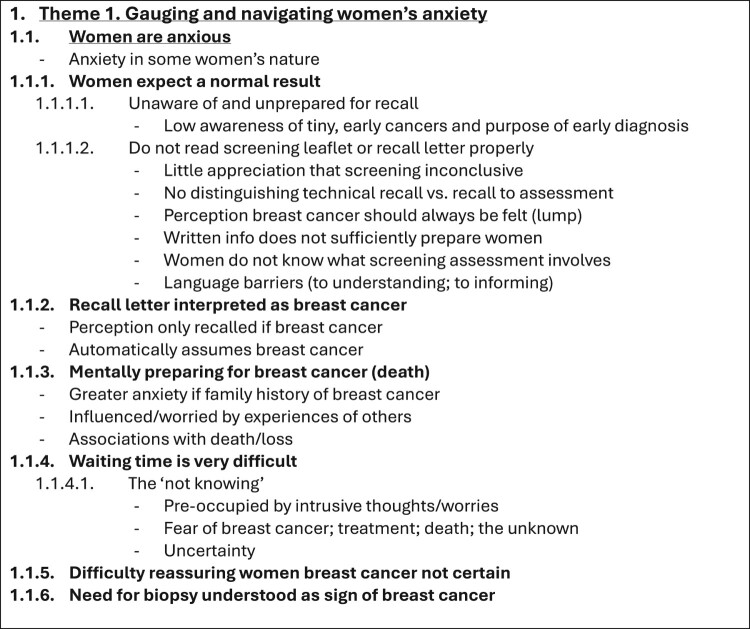
An example section of the first near-final Theme 1, with the overarching theme name, subthemes, and all relevant individual codes. The full version can be found in archived file 18 *Template 5_longer* on the OSF and ReShare. While it may seem that the template structure has shortened and simplified over time (e.g. since Figure 2), it is not the case. With close examination and analysis of the data, and continued discussion among the study team, the coding increased in depth, representing increased discrimination and clarity in our thinking behind the data. Template 5 shows the first ‘top level’ theme (‘Gauging and navigating women’s anxiety’) for the final version of our template.

**Figure 4.**
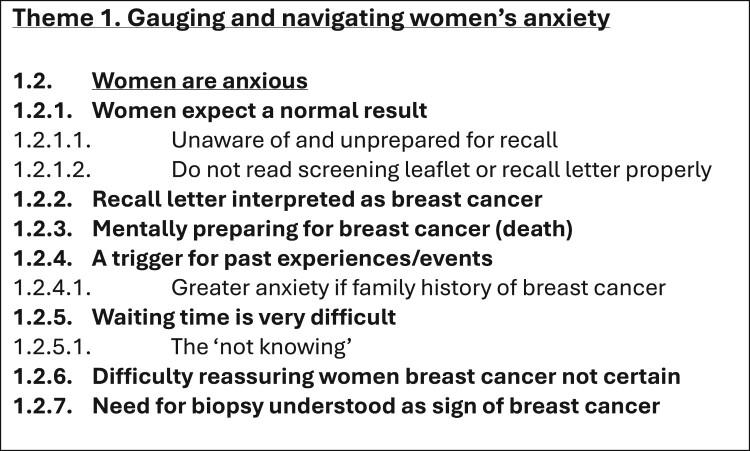
An example section of Theme 1 in the final template. The full version can be found in archived file 20 *Template 7* on the OSF and ReShare. This template structure is again shorter than the previous structures. Depth (rather than breadth) of coding allows fine distinctions to be made in key areas; having too many themes and subthemes makes it difficult for the researcher to draw together the analysis as a whole (Brooks et al., 2015).

The course of analysing the interview transcripts (i.e. raw data) produced various documents (i.e. files depicting processed data). Therefore, these processed data files were created in addition to and separately from the raw data files. Specifically, as the interview transcripts underwent coding, these codes and initial themes were recorded and organised in separate documents outside of the interview transcript files. In time, the iterative analysis and development of codes, themes, and thematic structures resulted in several versions of the template (depicted across several processed data files). Therefore, the processed data files stored on the OSF (Long et al., 2019a) and Reshare (UK Data Service) (Long et al., 2024a) show the sequential development of the analysis. Table 2 provides a chronological list of the 20 data analysis files generated in this study (available on the OSF (Long et al., 2019a) and ReShare (Long et al., 2024a)) and the steps of template analysis to which these files relate. The creation of these files is described in the following sections. Figures 1–4 depict illustrative examples of the steps of developing the first Theme using template analysis (from preliminary coding, devising the initial template structure, and to the final template structure). Table 3 provides a list of data files that were not archived and are therefore not publicly available.

**Table 2.** Data files archived for access via the Open Science Framework and ReShare.

File no.	Analysis stage	File label	File type	Data	Level of processing	Level of access
1	Preliminary coding: preliminary coding of the data was carried out. This is essentially the same process as used in most thematic analysis approaches, whereby the researcher begins by highlighting anything in the text that might contribute toward their understanding, and which appears relevant to the analysis.	Preliminary codes 1	Microsoft Word (.docx)	Codes	4 – thematic or topical analysis (codes)	A – open
2	Preliminary coding	Preliminary codes 2	Microsoft Word (.docx)	Codes	4 – thematic or topical analysis (codes)	A – open
3	Clustering codes and developing the initial template: the emerging themes are organised into meaningful clusters to begin to define how they relate to each other within and between these groupings.	Clustering/Template 1	Microsoft Word (.docx)	Codes	4 – thematic or topical analysis (codes)	A – open
4	Development of initial template: it is normal in template analysis to develop an initial version of the coding template on the basis of a subset of the data (rather than carrying out preliminary coding and clustering on all accounts before defining the thematic structure).	Template 2	Microsoft Word (.docx)	Template, including codes, subthemes and themes	4 – thematic or topical analysis (codes, subthemes, themes)	A – open
5	Development of initial template	Template 3 shorter (2021)	Microsoft Word (.docx)	Template, including codes, subthemes and themes	4 – thematic or topical analysis (codes, themes, subthemes)	A – open
6	Development of initial template	Template 3 longer (2021)	Microsoft Word (.docx)	Template, including codes, subthemes and themes	4 – thematic or topical analysis (codes, subthemes, themes)	A – open
7	Further coding as per row 1 above	New codes February 2023	Microsoft Word (.docx)	Codes	4 – thematic or topical analysis (codes)	A – open
8	Development of initial template	Template 3 longer_revised (2023)	Microsoft Word (.docx)	Template, including codes, subthemes and themes	4 – thematic or topical analysis (codes, themes, subthemes)	A – open
9	Applying, modifying, and developing the template: the initial template is applied to further data and modified as necessary. New themes, subthemes, and codes may be added and existing themes redefined or deleted if they seem redundant.	Template 3.1.	Microsoft Word (.docx)	Template, including codes, subthemes and themes	4 – thematic or topical analysis (codes, themes, subthemes)	A – open
10	Applying, modifying, and developing the template	Template 3.2.	Microsoft Word (.docx)	Template, including codes, subthemes and themes	4 – thematic or topical analysis (codes, themes, subthemes)	A – open
11	Applying, modifying, and developing the template	Template 3.3.	Microsoft Word (.docx)	Template, including codes, subthemes and themes	4 – thematic or topical analysis (codes, themes, subthemes)	A – open
12	Applying, modifying, and developing the template	Template 3.4.	Microsoft Word (.docx)	Template, including codes, subthemes and themes	4 – thematic or topical analysis (codes, themes, subthemes)	A – open
13	Applying, modifying, and developing the template	Template 4	Microsoft Word (.docx)	Template, including codes, subthemes and themes	4 – thematic or topical analysis (codes, themes, subthemes)	A – open
14	Applying, modifying, and developing the template	Template 4 into Template 5	Microsoft Word (.docx)	Template, including codes, subthemes and themes	4 – thematic or topical analysis (codes, themes, subthemes)	A – open
15	Applying, modifying, and developing the template	Template 5 shorter_temporary	Microsoft Word (.docx)	Template, including codes, subthemes and themes	4 – thematic or topical analysis (codes, themes, subthemes)	A – open
16	Applying, modifying, and developing the template. This file is annotated with comments and thinking behind changes to the template structure.	Template 5 longer_temporary_annotated	Microsoft Word (.docx)	Template, including codes, subthemes and themes	4 – thematic or topical analysis (codes, themes, subthemes)	A – open
17	Applying, modifying, and developing the template	Template 5 shorter	Microsoft Word (.docx)	Template, including codes, subthemes and themes	4 – thematic or topical analysis (codes, themes, subthemes)	A – open
18	Applying, modifying, and developing the template	Template 5 longer	Microsoft Word (.docx)	Template, including codes, subthemes and themes	4 – thematic or topical analysis (codes, themes, subthemes)	A – open
19	Final template version and interpretation: finalise the template and apply it to the full data set.	Template 6	Microsoft Word (.docx)	Template, including codes, subthemes and themes	4 – thematic or topical analysis (codes, themes, subthemes)	A – open
20	Final template version	Template 7	Microsoft Word (.docx)	Template, including codes, subthemes and themes	4 – thematic or topical analysis (codes, themes, subthemes)	A – open

Data files 1–20 are available on the OSF (Long et al., 2019a) and ReShare (UK Data Service) (Long et al., 2024a). We have described the steps of template analysis (Brooks et al., 2015) in relation to these files. We have adopted the UK Data Service’s access levels and conditions.’ (n.d.a.)

**Table 3.** Closed data files that are not archived.

Data	File type	Level of processing	Level of access	Additional consent to consider
12 audio files of interviews	Audio file (.wav)	0 – raw data, all identities included	C – closed; controlled by data access committee	Interviewer
12 interview transcripts from professional transcriber	Microsoft Word (.docx)	0 – raw data, all identities included	C – closed; controlled by data access committee	Transcriber and interviewer
12 de-identified and pseudonymised interview transcripts from professional transcriber	Microsoft Word (.docx)	1 – redaction of direct identifiers	C – closed; controlled by data access committee	Transcriber and interviewer
12 de-identified and pseudonymised interview transcripts from professional transcriber	Paper	1 – redaction of direct identifiers	Destroyed; no longer available	
Sample demographic questionnaire data	Microsoft Excel (.xl)	1 – raw data, redaction of direct identifiers	C – closed; controlled by data access committee	
Field notes	Paper notebook	1 – redaction of direct identifiers	C – closed; controlled by data access committee	Interviewer
Reflexive journal	Paper notebook	1 – redaction of direct identifiers	C – closed; controlled by data access committee	Interviewer
Reflexivity statement	Microsoft Word (.docx)	4 – topical analysis	A – open (published open access as Supplementary Information to study report) (Long et al., 2024b)	Interviewer
6 draft descriptions of the thematic template and analysis narrative with de-identified data excerpts as illustrative participant quotes	Microsoft Word (.docx)	3 – thematic aggregation with excerpted text	B – restricted	Interviewer

We have adopted the UK Data Service’s access levels and conditions, whereby ‘C – controlled access’ is managed by the researchers and only available to users who have been trained and accredited and their data usage has been approved by the relevant Data Access Committee.’ (n.d.a.)

### Preliminary coding

Following guidance of template analysis authors (King & Brooks, 2017; King & Brooks, 2017), *preliminary coding* was performed on a specific subset of data. The aim was to ensure a diverse representation of participant experiences within this subset of transcripts, as the assigned codes served as the building blocks for the initial templates. Diversity was pragmatically achieved by coding a transcript from each of the main HCP roles sampled: breast radiographer, advanced radiographer practitioner, breast radiologist, and clinical nurse specialist (a total of 4 transcripts). Further, HAL's understanding of the breadth of participants’ data, facilitated through data familiarisation (King & Brooks, 2017), enabled the selection of additional variation within the transcripts, based on the views and experiences participants shared.

The coding process was inductive (data-driven) whereby segments of the data deemed meaningful or relevant to the research aim were highlighted and subsequently labelled with descriptive and analytical codes (King & Brooks, 2017; King & Brooks, 2017). HAL conducted the first round of coding on printed transcripts. Further reflections and ideas for potential underlying meanings were recorded in the page margins. Subsequently, a second round of coding was undertaken on ‘clean’ digital copies of the transcripts in Microsoft Word (Figure 1). This step facilitated HAL to review, re-examine, compare, and contrast codes assigned on the printed transcripts against codes on their digital counterparts. Resulting codes were transferred to a Microsoft Word document, accessible in the data file 1 *Preliminary codes 1.*

Following this, HAL refined existing codes and introduced new codes. This process was iterative; HAL moved continuously back and forth between the four transcripts to create and refine codes as data were analysed. For example, the code ‘imagines the worst’ was deemed to be adequately captured by the broader code ‘women automatically assume/imagine breast cancer’. After several iterations, the preliminary list of codes was further processed, organised, and reduced in quantity (from 404 codes to 332 codes). The revised list of codes is available in the data file 2 *Preliminary codes 2*.

### Clustering codes and developing an initial coding template

To facilitate a meeting, HAL loosely organised codes by clustering these based on their similarities and their relevance to the study aim. HAL and JMB met to discuss the preliminary list of codes and initial groupings. JMB is an experienced academic qualitative psychologist with expertise in template analysis. Together, they began organising the codes into meaningful clusters based on how they related to other codes within (hierarchically) and between (laterally) clusters. Tentative patterns in the data were identified and proposed. The output of this meeting is available in the data file 3 *Clustering_Template 1*, which provides a broad, ‘big picture’ perspective of the analysis, highlighting initial clusters of codes in a temporary thematic arrangement, while not yet encompassing the entire list of codes.

### Development of initial template: phase 1

Following this discussion, HAL progressed the analysis by refining and formalising the clusters into a preliminary coding template, denoted as *Template 2* in data file 4. Further analysis involved a comprehensive review of the coded transcripts alongside the template. Adjustments were made to the placement and naming of codes, subtheme levels, and themes. Three overarching themes and various subtheme levels were created.

To facilitate a study team meeting, HAL produced an accompanying narrative written at theme and subtheme level. This document described and defined the preliminary themes. Additional changes to the template structure were introduced in the process to assist narrative flow. This led to the generation of a detailed version and a concise version of the template: data file 5 *Template 3 shorter (2021)* and 6 *Template 3 longer (2021)*. The longer version captured every code under the corresponding subtheme levels, preserving the intricacies and subtleties of the data. The shorter version, a succinct ‘quick look’ rendition, was designed to illustrate the primary theme and subtheme level structure with the aim of facilitating and supporting the team discussion.

### Development of initial template: phase 2

At this point, data analysis was suspended due to a leave of absence from work by HAL. Analysis resumed two years later, during which time the study team discussed how to continue the analysis while maintaining its integrity. A notable advantage of template analysis is its comprehensive audit trail, which clearly outlines the processes and decisions undertaken (King & Brooks, 2017). Upon reflection, Template 3 was a useful version with which to pause and resume analysis. This template, while detailed and thorough, was relatively underdeveloped. Its three themes were viewed more as tentative, descriptive placeholders rather than as robust, analytical representations of the data. Structurally, the template was somewhat flat and bottom-heavy with codes, lacking a compelling thematic hierarchy that meaningfully addressed the research aim. HAL undertook a meticulous review of all transcripts, lists of codes, and template versions to facilitate data re-familiarisation. HAL closely reviewed the previously coded subset of transcripts and deliberately re-coded these transcripts anew in order to record areas of agreement and disagreement. Approximately 40 new codes were created (data file 7 *New codes February 2023*), while approximately 35 former codes were discontinued, based on new or revised interpretations of the data (evident in data file 8 *Template 3 longer_revised (2023)* and Figure 2). HAL also revisited an existing 4000-word narrative (originally written to accompany Template 3), annotating it with new ideas and interpretations of the data.

### Applying, modifying, and developing the template

Between Template 3 and 4, substantial revisions were made to enhance the structural integrity of the template and refine thematic distinctions. The latest Template 3 version (featuring the new codes) was applied to four additional transcripts, each representing diverse healthcare professional roles. To ensure comprehensive coding of the new data, the iterative process involved the creation of new codes, amendments to existing codes, and the consolidation of similar codes. Template 4 evolved through a series of alterations to codes, subtheme levels, and themes (documented across data files 9-13: *Template 3.1, Template 3.2, Template 3.3, Template 3.4* and *Template 4*). This version of the template retained three primary themes and several subtheme levels, with a refined analytical ‘story.’ An accompanying narrative was developed to describe and define the themes and subthemes, and included data excerpts as illustrative quotes. Both the template and narrative were discussed by the study team. The study team generally agreed upon two themes, although certain subthemes were refined and clarified. The third theme was deemed somewhat vague and it was agreed that this theme possessed elements that were potentially applicable to other themes. The study team decided to integrate these elements into the relevant themes, leading to the creation of Template 5 (data file 14 *Template 4 into Template 5*), which was then ‘tested’ on additional new data.

Consequently, HAL created a temporary Template 5 and applied it to two additional transcripts (data file 15 *Template 5 shorter_temporary*). Further coding was undertaken alongside structural template alterations. The template was annotated with new ideas and connections (data file 16 *Template 5 longer_temporary_annotated*). Subsequently, HAL and JMB convened to discuss the template in depth and made further amendments to the structure and names of codes, subtheme levels, and overarching themes. The subthemes within the second theme were reordered to enhance narrative flow. Additionally, certain subtheme levels required a more nuanced delineation to comprehensively capture the range of participants’ experiences and perspectives. HAL applied the template to an additional transcript and made minor changes to the structure. Following these edits, HAL and JMB met again to apply the work-in-progress Template 5 to the final transcript, resulting in a new subtheme level. The study team discussed Template 5 and an accompanying narrative and agreed it as the near-final template (data file 17 *Template 5 shorter* and 18 *Template 5 longer –* Figure 3).

### Final template version and interpretation

Template 5 was applied to all transcripts again. This was conducted in Microsoft Word. Only minor changes were made, and a near-near-final template was created (data file 19 *Template 6*). A revised narrative accompanying Template 6 was produced. The template structure and study findings were agreed by the study team.

The template underwent additional amendments while preparing the study manuscript for publication. Specifically, there was a particular subtheme that initially seemed relevant to the study aim. Upon consideration, it became apparent that the findings within this subtheme did not offer novel insights beyond what is already documented in existing literature. Therefore, to maintain focus on the original and unique contributions of the study, this subtheme was omitted from the final template. Accordingly, this is reflected in the final and published template (data file 20 *Template 7* and Figure 4). The final few revisions of the Results were agreed by all authors.

## Discussion

The present data note describes 20 data files generated in the template analysis of a qualitative interview study with breast screening healthcare professionals (Long et al., 2024b). The files provide context and detail about the template analysis procedure and outputs and are intended to benefit researchers in disciplines such as psychology, health services research, cancer screening, and public health.

It is important to have exemplars of qualitative data analysis. Offering clear, detailed, and openly accessible accounts of the data analysis process can serve as a valuable resource (particularly in cases where the raw dataset is closed due to participant identifiability) by allowing other researchers to understand the process in depth. This approach fosters research transparency. It is in contrast to the predominant research article format, which involves reporting the final analysis template only, usually due to publisher constraints and reporting conventions. Thus, the present level of transparency is often lacking in qualitative health research, yet can be useful for understanding the methodology, analysis and study results, while simultaneously demonstrating and advancing open qualitative research practices. Further, for those new to qualitative analysis, a worked example serves as a valuable resource, guiding them through the complexities of the analysis process.

### Limitations

While there are legitimate reasons why the raw data are closed, it is a limitation that the processed data described in this data note cannot be understood alongside the interview transcripts. It has meant the processes and decisions described are necessarily divorced from the raw data upon which they were based, which may hinder understanding. One key advantage of template analysis is its flexibility and adaptability. However, this flexibility can also be a disadvantage when it comes to reporting analytical procedures. Similar to many qualitative approaches, template analysis relies on the researcher’s interpretive judgement and numerous, cumulative decisions made throughout analysis. This can make it challenging to articulate the process in a way that is meaningful, instructive, and genuinely useful to others. However, by providing detailed documentation of our procedures, we hope that this data note demonstrates a possible approach to addressing these challenges, offering a guide for researchers who may wish to adopt similar practices. In the process, we have demonstrated a means of engaging with open qualitative research practices in circumstances where it is not possible to make a dataset publicly available.

Additionally, it is a limitation of our study that more of our processed data files and materials are not publicly available. It is worth acknowledging that we did not embark on our qualitative interview study (Long et al., 2024b) with the view to create an archive of our processed data files and materials. As such, several processed data files are not available to share (Table 3), including, for example, documents describing and defining themes and subthemes. These files included narrative summaries of the data and our initial insights, which were instrumental in shaping our discussions and data interpretation during study team meetings. These files offered a richly detailed picture of the progression of themes. However, these files are not publicly available due to concerns about participant identifiability with regards to their nature as work-in-progress materials. While we have endeavoured to share as much of our process in our data archive and the present data note, the unanticipated decision to make materials openly available after study completion limited our ability to plan for greater transparency from the outset. This reflects a broader challenge in qualitative research regarding balancing openness with the need for creative and iterative analysis, allowing room for ‘error’ and evolving ideas.

To the best of our knowledge, there are no established data note templates or guides, or publisher guidelines specifically for data notes describing qualitative data, whether raw or processed. Current data note guidelines are predominantly designed for quantitative datasets. Therefore, we have endeavoured to make this data note as comprehensive and instructive as possible for our readers.

## Data Availability

The processed data (i.e. all 20 data analysis files reported in the present data note) are available on the OSF (Long et al., 2019a) and ReShare (UK Data Service) (Long et al., 2024a). Highly identifiable data (i.e. participants’ interview audio-recordings and transcripts) and data in a physical format (i.e. field notes) are only available to the study team.
